# Much Lower Prevalence and Mortality of Chronic Obstructive Pulmonary Disease in Japan Than in the United States Despite Higher Smoking Rates: A Meta-Analysis/Systematic Review

**DOI:** 10.2188/jea.JE20240085

**Published:** 2025-02-05

**Authors:** Akira Sekikawa, Mengyi Li, Niva Joshi, Brandon Herbert, Curtis Tilves, Chendi Cui, Shiyao Gao, Yuefang Chang, Yasutaka Nakano, Frank C Sciurba

**Affiliations:** 1 Department of Epidemiology, School of Public Health, University of Pittsburgh, Pittsburgh, PA, USA; 2 School of Medicine, University of Pittsburgh, Pittsburgh, PA, USA; 3 Division of Respiratory Medicine, Department of Internal Medicine, Shiga University of Medical Science, Shiga, Japan

**Keywords:** COPD, epidemiology, Japan, and the US, mortality, prevalence, smoking

## Abstract

**Background:**

A recent systematic review showed Japan’s mortality from chronic obstructive pulmonary disease (COPD) is the lowest among 204 countries, despite notably higher smoking rates in men in Japan than in the United States. This study aims to compare (1) trends in smoking rates, (2) trends in COPD mortality, and (3) the spirometry-based COPD prevalence in the general adult population between Japan and the United States.

**Methods:**

Age- and sex-specific smoking rates from the 1980s through 2010s and COPD mortality from 1999 through 2019 were obtained from national surveys and official statistics (International Classification of Diseases-10^th^ codes J40–44), respectively. A systematic review and meta-analysis was performed to estimate COPD prevalence in Japan, while the National Health and Nutrition Examination Survey 2007–2012 was used for the United States. A fixed ratio of 0.7 of forced expiratory volume in the first second of forced vital capacity was used to define COPD.

**Results:**

Over the past 4 decades, men in Japan consistently had 20–30% higher smoking rates than their United States counterparts. From 1999–2019, age-adjusted COPD mortality in men in Japan was only a third of the United States, whereas that in women was less than a tenth in 2019. Synthesizing data from 11 studies, involving 89,955 participants, Japan’s COPD prevalence was more than 10% lower than in the United States in almost all age groups for both sexes.

**Conclusion:**

This study showed markedly lower rates of COPD in Japan than in the United States. Investigating factors contributing to the paradoxical observations could lead to advancing COPD risk reduction strategies.

## INTRODUCTION

Chronic obstructive pulmonary disease (COPD) is characterized by persistent respiratory symptoms, such as dyspnea, cough, and excessive sputum production, accompanied by incompletely reversible airflow obstruction.^1^ COPD is recognized as a multifaceted respiratory ailment with contributions of airway narrowing and parenchymal emphysema varying between individuals and associated with extra-pulmonary comorbidities, including cardiovascular disease (CVD).^2^ In 2019, it ranked the fourth leading cause of death in the United States^1^ and the third leading cause worldwide.^3^

A prominent risk factor for COPD in developed countries is smoking.^4^ Notably, Japan exhibits markedly higher smoking rates than other developed countries, especially in men. A systematic review in Japan reported a strong relationship between smoking and COPD in this population: the odds ratio (OR) for COPD was 3.57 in current smokers compared to non-smokers.^5^ Intriguingly, despite this, the Global Burden of Disease Study 2019 revealed Japan to have the lowest mortality from COPD among 204 countries.^3^

COPD frequently goes undiagnosed, with estimates indicating that 80% of affected individuals in Japan remain undetected.^6^ This estimate is drawn from several population-based studies among adults where spirometry is performed for all participants without screening. These studies indicate that the prevalence in adults aged ≥40 years in Japan is approximately 10%, which aligns with global prevalence figures reported in a systematic review published in 2006.^7^

Spirometry is the gold standard for diagnosing COPD, as emphasized by the Global Initiative for Chronic Obstructive Lung Disease (GOLD) guidelines, which advocate for the use of a fixed ratio of 0.7 of the forced expiratory volume in the first second of the forced vital capacity (FEV_1_/FVC).^8^ Since the Japanese Respiratory Society issued recommendations for the standardization of spirometry in 2004, numerous epidemiological studies in Japan have employed spirometry on entire adult participants, eschewing pre-screening. This stands in contrast to the United States, where population-based spirometry screening is not recommended. However, the National Health and Nutrition Examination Survey (NHANES) performed spirometry tests on American adults.

This study aims to compare Japan and the United States; first, for the trends in smoking rates, and second, for the trends in COPD mortality. Finally, we aim to compare the spirometry-based COPD prevalence in the general adult population between Japan and the United States by estimating COPD prevalence with a systematic review and meta-analysis in Japan and the data from the NHANES in the United States.

## METHODS

The present work utilized the National Health and Nutrition Survey in Japan, the NHANES, the National Health Interview Survey (NHIS) from the National Center for Health Statistics, and the Centers for Disease Control and Prevention Wide-Ranging Online Data for Epidemiologic Research (CDC WONDER) database in the United States and World Health Organization (WHO) mortality database to obtain rates of current smokers and COPD mortality in Japan and the United States. The present systematic review and meta-analysis was conducted to determine the prevalence of COPD in Japan and followed the recommendations of the Preferred Reporting Items for Systematic Reviews and Meta-Analysis.^9^ The study protocol was registered on the PROSPERO International Prospective Register of Systematic Reviews (CRD: 42016041408). Institutional Review Board permission and informed consent were not required to conduct this trial, as this study did not involve human subjects.

### Rates of current smokers in Japan and the United States

Age- and sex-specific smoking rates from the 1980s through 2010s in Japan were obtained from the National Health and Nutrition Survey.^10^ The rates in the United States were obtained from the NHIS from the National Center for Health Statistics.^9^

### COPD mortality in Japan and the United States

The WHO mortality database from 1999 to 2019 was used to synthesize age- and sex-specific COPD mortality in Japan. For the United States, the WHO mortality database was used for the years 1999 to 2006, and CDC WONDER database for the years 2007 to 2019 because the WHO mortality database for the United States was updated through 2006 only. COPD death was defined as the underlying cause of death under the 10^th^ Revision of the International Classification of Disease (ICD-10) codes J40–J44. The year 1999 was chosen because the ICD-10 was implemented for mortality coding from death certificates in the United States in 1999. The year 2019 was chosen to describe the mortality before the start of the coronavirus disease 2019 pandemic. COPD mortality rates were calculated by using the number of COPD deaths among age groups of 45–54, 55–64, 65–74, and ≥75 years, divided by the population of that equivalent age group and expressed per 100,000.

### Prevalence of COPD in Japan

A meta-analysis was conducted to estimate the age- and sex-specific prevalence of COPD in Japan. The following inclusion criteria were applied: 1) English-language publication only; 2) participants above 18 years old; 3) observational studies conducted in Japan; 4) pulmonary function test by spirometry was performed without screening; 5) COPD was defined as FEV_1_/FVC <0.7; and 6) age- and sex-specific data on COPD prevalence was available. PubMed, Embase, and Cochrane database were searched comprehensively for publications from January 1995 through January 2022. The complete search strategies are described in eTable 1.

**Figure 1.**
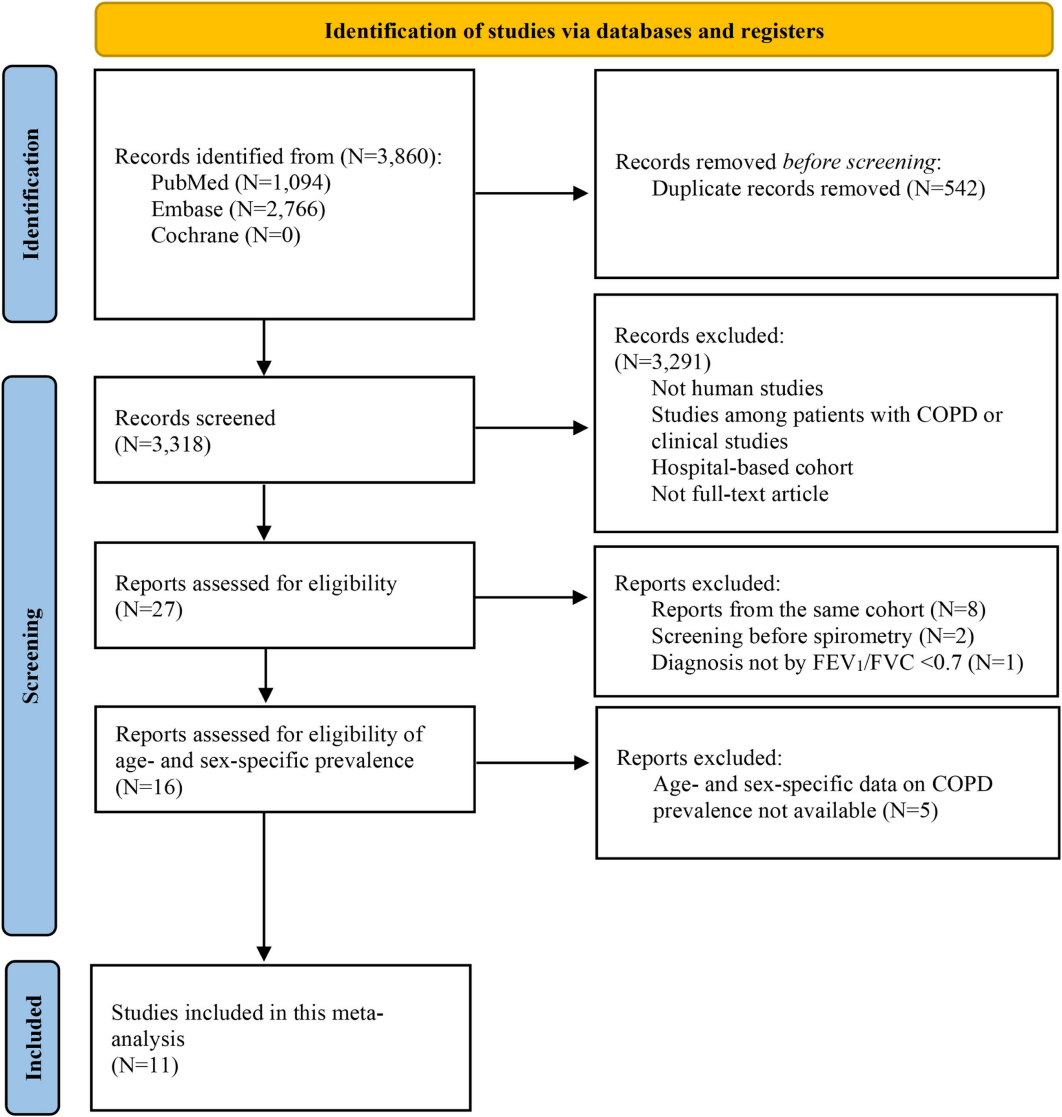
PRISMA flow diagram for new systematic reviews which included searches of databases and registers only. COPD, chronic obstructive pulmonary disease; FEV_1_/FVC, forced expiratory volume in the first second of the forced vital capacity; PRISMA, Preferred Reporting Items for Systematic Reviews and Meta-Analyses.

A review of titles, abstracts, and full texts of each article was conducted by five reviewers (NJ, CC, CT, SG, and AS). First, titles/abstracts that did not meet inclusion criteria were screened out. Second, full texts of the remaining articles were thoroughly reviewed and were excluded if they did not meet the inclusion criteria. Any disagreements during the study selection were resolved by discussion among reviewers (NJ, ML, CT, CC, SG, and AS). We identified 16 studies that met our inclusion criteria. If an article did not present the age- and sex-specific prevalence of COPD (*n* = 12), we contacted the author and requested the data. Out of 12 such studies, 7 provided the data, resulting in a total of 11 studies included for this meta-analysis of COPD prevalence (Figure 1). The quality of studies included in this meta-analysis was evaluated by the investigators (NJ, ML, and AS) using the Joanna Briggs Institute checklist.

### Prevalence of COPD in the United States

To estimate the prevalence of COPD in the United States, we combined data from three NHANES cycles between 2007–2012, when the spirometry test was performed among adults of the United States population aged 40–79 years. The NHANES employs a complex, multistage sampling methodology, designed to ensure that the survey sample is representative of the United States population.^10^ Among 10,219 participants aged 40–79 years, 8,002 completed the spirometry. We excluded those with self-reported emphysema or chronic bronchitis (*n* = 569), resulting in 7,433 participants. We used a pre-bronchodilator value of FEV_1_/FVC <0.7 to diagnose COPD to compare the COPD prevalence between the United States and Japan, since almost all studies in Japan used a pre-bronchodilator value of FEV_1_/FVC.

### Statistical analysis

To estimate the prevalence of COPD in the United States from the combination of three NHANES cycles, we divided each participant’s survey weight by 3, per NHANES analytics protocol. All analyses of NHANES data used complex survey procedures in SAS 9.4 (SAS Institute, Cary, NC, USA). To estimate age-adjusted COPD mortality, we employed the direct age-adjustment method using the United States 2000 census population as the standard population. Age- and sex-specific pooled prevalence of COPD was calculated with ‘meta’ package (version 6.5.0) in R (Version 4.2.3; R Foundation for Statistical Computing, Vienna, Austria). A generalized linear mixed model framework with random intercept for logit transformed proportions was used for meta-analysis to account for between and within study uncertainties. I^2^ was used to assess the heterogeneity of age-specific COPD prevalence. A *P*-value of <0.05 is considered statistically significant.

## RESULTS

**Figure 2.**
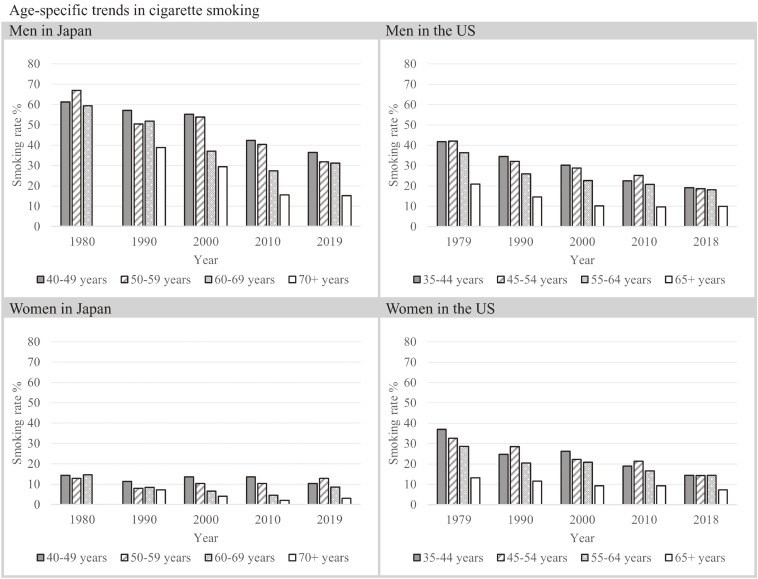
Age- and sex-specific trends in cigarette smoking in Japan and the United States

In men in Japan and the United States, the rates of current smokers have been decreasing from 1980 through 2019 in every age group. However, the rates were consistently higher in Japan than in the United States by 20–30% for each decade and in all age groups (Figure 2). In women in Japan, the rates of current smokers were consistently lower than in men in Japan (Figure 2), and the rates remained 2–15% during this period.

### COPD mortality

Both in Japan and the United States, age-adjusted mortality from COPD decreased from 1999 to 2019 in both sexes. Age-adjusted mortality remained substantially lower in Japan than in the United States. In 1999, age-adjusted COPD mortality in men in Japan was about one third of that in the United States (52.9/100,000 vs 157.4/100,000, respectively) and in 2019, it was less than one third (35.8/100,000 vs 116.0/100,000, respectively). During this period, age-adjusted total mortality in men was lower in Japan than in the United States (4,648.3/100,000 vs 5,365.1/100,000 in 1999 and 3,445.3/100,000 vs 4,325.1/100,000 in 2019, respectively). The difference in age-adjust COPD mortality was more pronounced in women. In 1999, age-adjusted COPD mortality in women in Japan was less than one-eighth of that in the United States (12.5/100,000 vs 103.0/100,000, respectively) and in 2019, it was about one twentieth of that in the United States (5.2/100,000 vs 101.3/100,000, respectively). During this period, age-adjusted total mortality in women was lower in Japan than in the United States (2,686.8/100,000 vs 4,076.7/100,000 in 1999 and 2,274.8/100,000 vs 3,377.0/100,000 in 2019, respectively).

**Figure 3.**
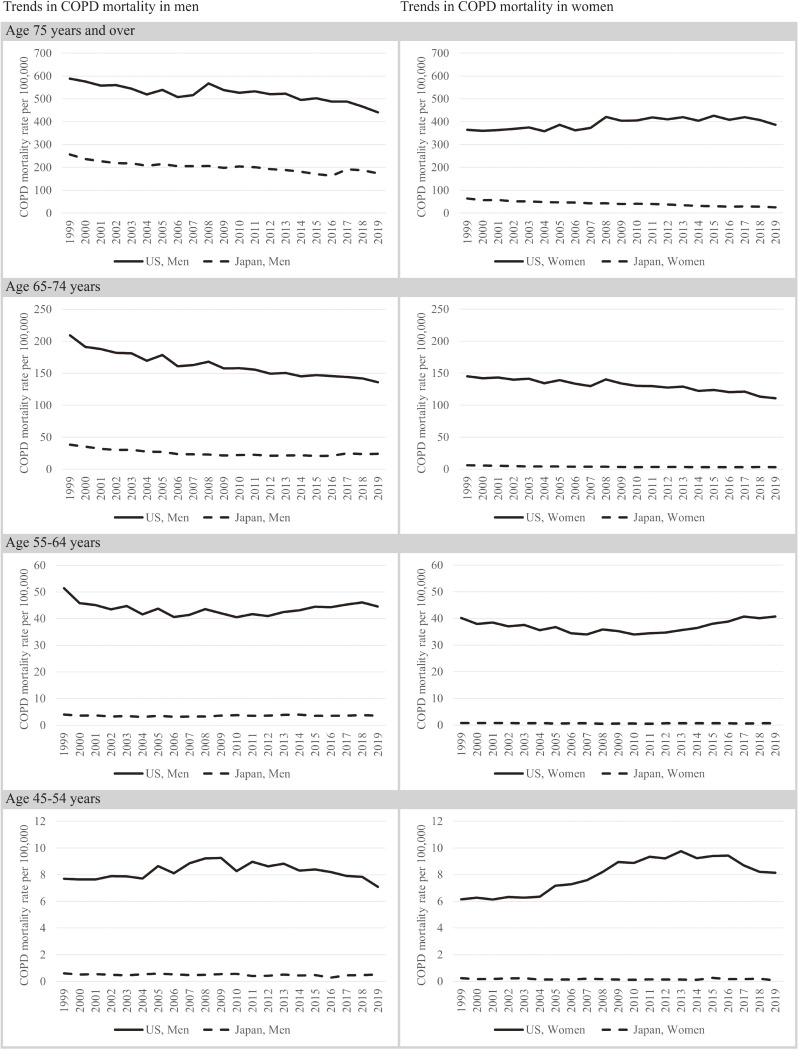
Age- and sex-specific trends in mortality from COPD in Japan and the United States. COPD, chronic obstructive pulmonary disease.

In men in Japan and the United States, COPD mortality in all age groups decreased from 1999 to 2019 (Figure 3). In women, COPD mortality in Japan fell from 1999 to 2019 in all age groups, whereas in the United States, it did not decrease much in women aged ≥75 years and it increased in women aged 45–54 and 55–64 years. The difference in age-specific COPD mortality between Japan and the United States was much more pronounced in women than in men.

### Meta-analysis of COPD prevalence in Japan

Characteristics of the 11 studies^11^^–^^21^ included in this meta-analysis are summarized in Table 1. The total number of participants was 89,955, ranging from 161 to 22,293, and 56% were men. Spirometry was conducted under standardized procedures by the American, European, or Japanese Thoracic Societies (Table 1). Several studies excluded participants with medically diagnosed COPD and other lung diseases. The quality of these included studies was generally good (eTable 2). Characteristics of five excluded studies^22^^–^^26^ (a total of 13,282 participants, with 48% men) were generally similar to these 11 included studies (eTable 3).

**Table 1.** Characteristics of the included studies for meta-analysis of COPD prevalence in Japan

Author, Year, reference	Number of participants (%)	Age, years, mean (range)	Population drawn	Use of broncho-dilator	FEV1/FCV <70%	Excluded from the study
Fukuchi 2004^11^	2,343 (52)	58.0 (40+)	Randomly selected from 18 prefectures	No	Standard procedures ATS/ERS	
Takemura 2005^12^	12,760 (68)	47.1 (30+)	Health checkups	No	Standard procedures ATS	Asthma, COPD other lung diseases
Omori 2007^13^	13,534 (63)	(40–69)	Health checkups	No	Standard procedures	Asthma, COPD, other lung diseases
Minakata 2008^14^	474 (56)	(40+)	Primary care clinic	No	Standard procedures ATS	Lung diseases
Osaka 2010^15^	2,917 (45)	62.8 (40+)	Community-based study	No	Standard procedures	
Horie 2013^16^	15,324 (63)	(30+)	School workers’ health checkups	No	Standard procedures ATS	
Azuma 2014^17^	303 (100)	43.9	Employees at a company	No	Standard procedures	Asthma
Fukutani 2015^18^	161 (48)	73 (65+)	Community-dwelling elderly	No	Standard procedures by JRS	Lung diseases
Omori 2016^19^	22,293 (63)	54.7 (40+)	14 centers in Japan	No	Standard procedures ATS/ERS	
Utsugi 2016^20^	950 (50)	64.9 (40–79)	13 Primary care clinics	Yes	Standard procedures by ATS	Lung diseases
Omori 2017^21^	9,896 (78)	49 (35–60)	5 Healthcare center	No	Not reported	Lung diseases other than asthma and COPD

ATS, American Thoracic Society; COPD, chronic obstructive pulmonary disease; ERS, European Respiratory Society; FEV_1_/FVC, forced expiratory volume in the first second of the forced vital capacity; JRS, Japanese Respiratory Society.

### Prevalence of COPD in Japan and the United States

**Figure 4.**
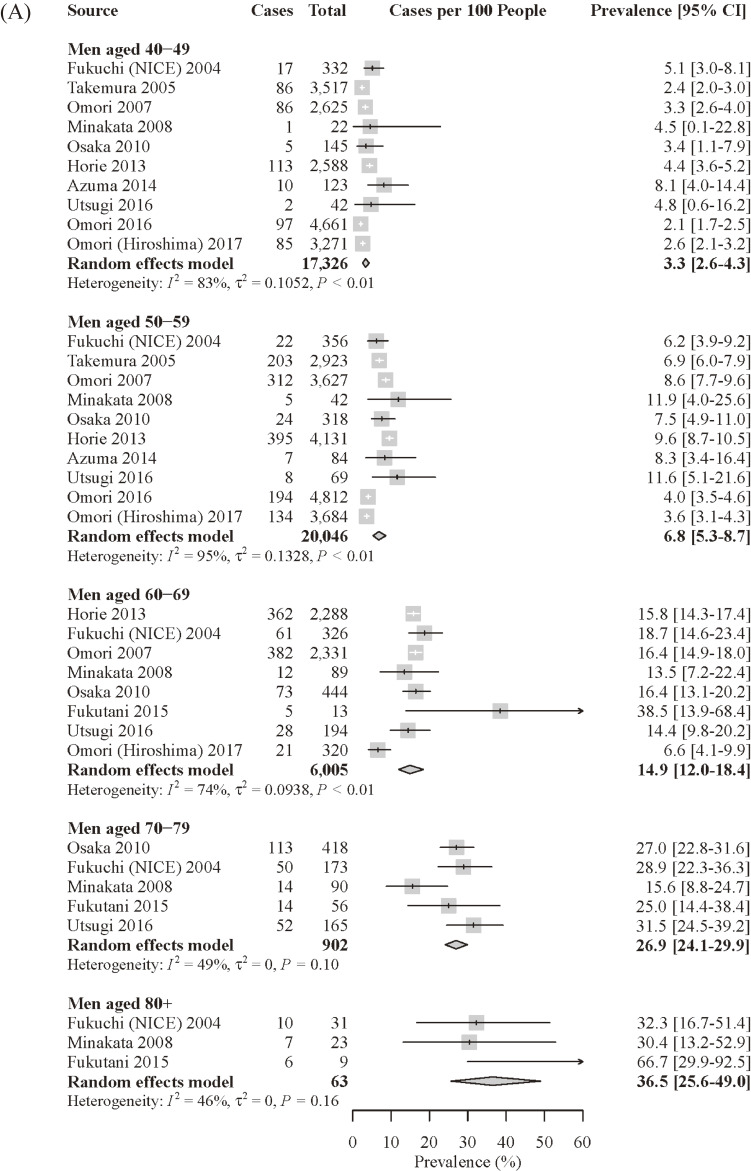

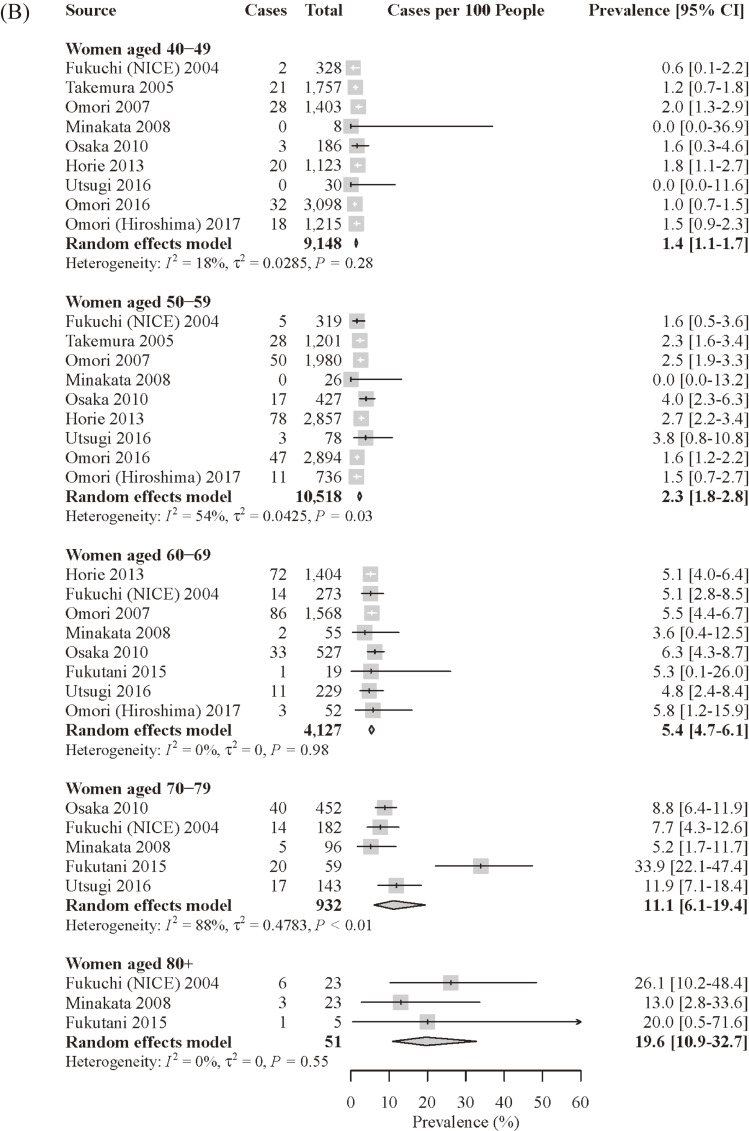
Age- and sex-specific prevalence of COPD in Japan by the meta-analysis; (**A**) age-specific prevalence of COPD in men in Japan; (**B**) age-specific prevalence of COPD in women in Japan. CI, confidence interval; COPD, chronic obstructive pulmonary disease.

In both sexes in Japan, the prevalence increased as age increased. The prevalence was higher in men than in women in all age strata. The prevalence in men for those aged 40–49, 50–59, 60–69, and 70–79 years was 3.3%, 6.8%, 14.9%, and 26.9%, respectively (Figure 4A). The prevalence in women was 1.4%, 2.3%, 5.4%, and 11.1%, respectively (Figure 4B).

**Figure 5.**
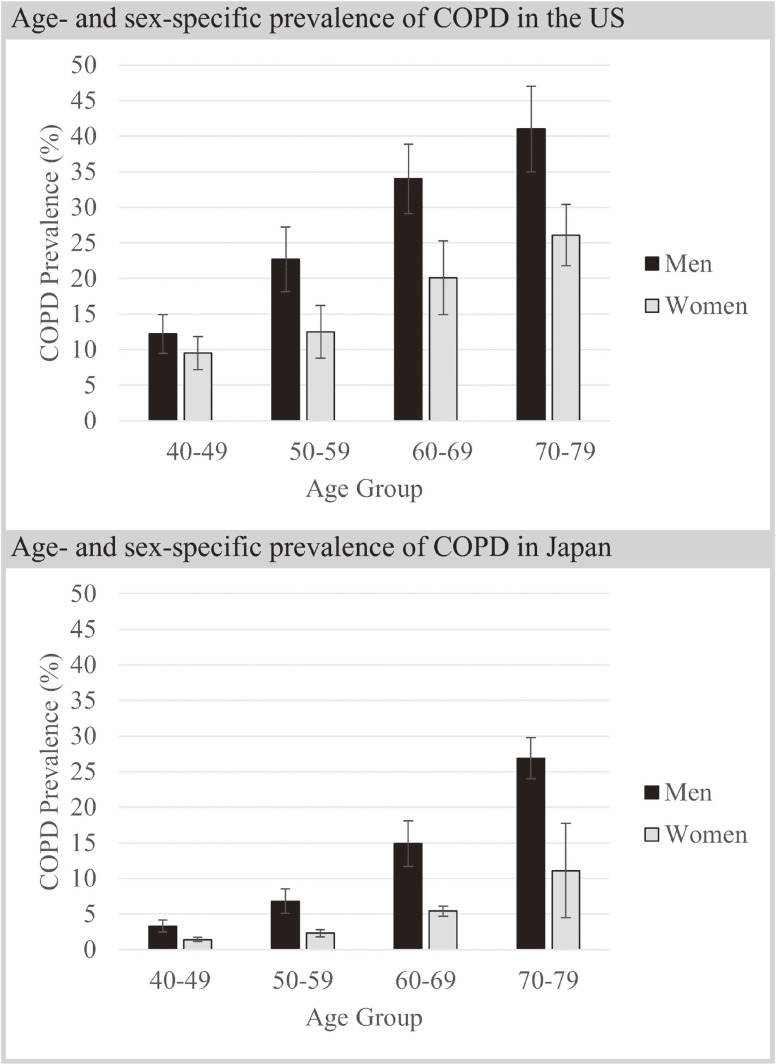
Age- and sex-specific prevalence of COPD in the United States from NHANES and in Japan based on meta-analysis. Bars represent 95% confidence interval. COPD, chronic obstructive pulmonary disease; NHANES, National Health and Nutrition Examination Survey.

In the United States, the prevalence was higher in men than in women in all age strata (Figure 5). The prevalence in men for those aged 40–49, 50–59, 60–69, and 70–79 years was 12.2%, 22.7%, 34.0%, and 41.0%, respectively. The prevalence in women was 9.5%, 12.5%, 20.1%, and 26.1%, respectively. Therefore, the prevalence of COPD in Japan was more than 10% lower than that in the United States both in men and women aged 50–59, 60–69 and 70–79 years.

## DISCUSSION

This is the first study to compare COPD prevalence using the same diagnostic criteria in the general population in Japan and the United States that also includes trends in mortality and current smoking rates. Notably, despite consistently higher smoking rates in Japanese men compared to American men, the age-adjusted COPD prevalence and mortality were lower in Japan. The much lower smoking rates in Japanese women compared to American women was expectedly associated with less COPD rates in Japanese women. Over the past four decades, men in Japan consistently had 20–30% higher smoking rates than their American counterparts, although the rates have been declining in both countries. Age-adjusted COPD mortality in men in Japan was only a third of the United States rate, while that in women in Japan was less than a tenth of the United States rate in 2019. Prevalence of COPD increased with age in both sexes in both countries. Prevalence of COPD in Japan was more than 10% lower than in the United States in every age group in both sexes except for men aged 40–49 years.

Recent reviews reported several distinct characteristics of patients with COPD in Japan compared to Western countries^6^^,^^27^; Japan has lower rates of CVD and exacerbation. These characteristics may partly contribute to the low COPD mortality in Japan. Among patients with COPD in Japan, mortality from CVD is 11 to 12%,^28^ whereas 22 to 35% of COPD patients suffer CVD-related mortality in Western countries.^29^ Moreover, in the general population, we reported that mortality from coronary heart disease (CHD) is >67% lower in Japan than in the United States, despite a worse profile of lifetime exposure to many CVD risk factors in Japan than in the United States.^30^ Since CHD mortality in Japan continues to decline over the past 5 decades, whereas migrant studies of Japanese to the United States documented a dramatic rise in CHD rates, the low CHD mortality in Japan is unlikely due to genetic susceptibility. COPD is significantly associated with CHD independent of smoking or other risk factors.^29^^,^^31^ Such an association may be linked through shared inflammatory mechanisms.^32^^,^^33^ Studies including ours^34^ and others^35^ that compared biomarkers of inflammation uniformly reported that levels of inflammation are significantly and substantially lower in Japan than in the United States. Therefore, low levels of systemic inflammation in Japan may contribute to low rates of COPD in Japan.

A large randomized controlled trial that recruited 5,993 patients with COPD from 37 countries using the same standardized protocol showed that the annual exacerbation rate is lower in Japan compared to the entire study sample (0.61 vs 0.85, respectively).^27^ The low exacerbation rate in Japan may be due to better access to medical care and closer management of the patients, resulting in increased disease stability, which may partly be associated with lower COPD mortality in Japan.

Several studies included in this meta-analysis reported smoking status-specific prevalence of COPD.^11^^–^^13^^,^^16^ Odds ratios of the prevalence of COPD in current smokers compared to never smokers ranged from 2.1 to 3.8. These findings are consistent with the results of the Burden of Obstructive Lung Disease study, a population-based prevalence study of COPD with spirometry across 12 international sites.^36^ Although rates of current smokers in men in Japan were higher than in the United States, it is possible that pack-years of smoking are lower in Japan than in the United States. However, available evidence suggested the opposite. Hisamatsu et al compared the progression of coronary artery calcification from population-based samples of men from the Shiga Epidemiological Study of Subclinical Atherosclerosis in Japan (*n* = 697; mean age, 63 years) and the Multi-Ethnic Study of Atherosclerosis in the United States (*n* = 1,712; mean age, about 60 years).^37^ Pack-years of smoking in current and former smokers in men in Japan are significantly higher than in the United States by >10 pack-years.

It is possible that genetic factors contribute to the apparent lower susceptibility to COPD in Japanese compared to United States smokers or to differences in phenotypic expression and disease progression. Alpha-1 antitrypsin deficiency is one known, albeit minor genetic determinant of COPD. Because the frequency is very low, this would not explain the difference in the observed rates between populations. Several recent genome-wide association studies of COPD and lung function reported the ethnic difference including East Asians.^38^^,^^39^ However, the differences are trivial and seem unlikely to contribute to the large differences in rates of COPD. Similar migrant studies of Japanese to the United States performed with CHD would be necessary to convincingly show that the difference in rates of COPD is likely to be due to environmental exposure specific to Japan.

Among environmental factors, the Japanese diet is considerably different from diets in other developed countries. The Japanese diet is characterized by markedly high intake of long-chain n-3 polyunsaturated fatty acids (LCn-3PUFAs) from fish (its dietary intake in Japan is 10 times as high as in the United States^40^) and soy. LCn-3PUFAs have anti-inflammatory and pro-resolutory actions.^41^ Soy isoflavones, micronutrients contained in soy foods, are polyphenols and have strong anti-oxidant properties.^42^

Two large prospective cohort studies in the United States (the Nurses’ Health Study and Health Professional Follow-Up Study, *n* = 120,175) showed that dietary intake of fish was inversely associated with risk of COPD (adjusted-pooled Hazard Ratio [HR] for the highest compared to the lowest intake: 0.71; 95% CI, 0.54–0.94) after adjusting for age, sex, pack-years of smoking and other covariates.^43^ Moreover, the longitudinal National Heart, Lung, and Blood Institute Pooled Study (*n* = 15,063) reported a significant inverse association of LCn-3PUFAs, especially docosahexaenoic acid (DHA), with FEV_1_ (1.4 mL/year; 95% CI, 1.1–1.8 per 1% increase in DHA) and reduced risk of spirometry-defined airway obstruction (0.93; 95% CI, 0.89–0.97, per 1% increase in DHA) after adjusting for age, sex, smoking and other covariates.^44^ Additionally, a report from the United Kingdom Biobank, which followed 484,414 participants for 9 years, reported that fish oil supplementation is significantly and inversely associated with incident COPD after adjusting for other factors including smoking and comorbidity (HR 0.88; 95% CI, 0.84–0.93).^45^ A case-control study in Japan (*n* = 618) of COPD diagnosed within 2 years from Japan reported that dietary intake of soy isoflavones is significantly inversely associated with COPD (OR compared to the lowest to the highest quartile of soy isoflavones intake was 0.36; 95% CI, 0.19–0.68, *P* for trend = 0.034) after adjusting age, sex, smoking and other lifestyle factors.^46^

Other factors, such as differences in ambient air pollution levels or socioeconomic inequalities, might partly contribute to the observed dissimilarities in COPD rates between the two countries. Further, cultural differences in the interaction between patients and healthcare system could impact diagnosis, prescription of, or compliance with maintenance medications known to alter disease progression, exacerbation rate and mortality.^1^^,^^8^^,^^47^ Notably, while both prevalence and mortality exhibited marked diminution in Japan compared to the United States, the dissimilarity in mortality rates exceeded that of prevalence. This observation implies that the prognostic outlook for COPD in Japan might surpass that in the United States.

Limitations of this study warrant discussions. We used official statistics to compare COPD mortality, which has inherent limitations, including misclassification of causes and changes in coding systems. COPD deaths are often underreported or misclassified with diseases like pneumonia and heart disease in Japan,^48^ which could lead to COPD omissions. However, the study in the United States similarly reported COPD underreporting on death certificates.^49^ These findings across different countries underline the urgent need for enhanced death certification practices and further research to accurately reflect COPD’s impact in health statistics. We used codes only under ICD-10. In our sensitivity analysis, we added asthma (J45), but the results did not change materially (data not shown). Although age- and sex-specific COPD prevalence in Japan was estimated in the meta-analysis, there were variations in exclusion criteria among these studies. Considering that 80% of COPD is undiagnosed in Japan,^6^ the effect of the variations on estimated prevalence is not large. To estimate age- and sex-specific COPD prevalence in the United States using the NHANES dataset, we excluded self-reported emphysema or chronic bronchitis. Therefore, the actual prevalence is likely to be higher. We used the fixed ratio of FEV_1_/FVC in diagnosing COPD. This criterion tends to over-diagnose disease in older adults and underdiagnose disease in younger adults when compared with a population-derived, age-adjusted lower limit of normal.^50^ Nevertheless, we used the same diagnostic criteria to compare the prevalence in the general adult population without screening between Japan and the United States. Other strengths include that we presented age- and sex-specific data on smoking, mortality, and prevalence. Furthermore, we presented the trends in smoking since 1980, 20 years before the mortality data, considering the long incubation period of COPD.

In conclusion, this study showed markedly lower prevalence of and mortality from COPD in Japan than in the United States, especially in men, despite much higher rates of smoking in men in Japan. Investigating the factors contributing to the lower rates of COPD in Japan could lead to advancing COPD risk reduction strategies.

## SUPPLEMENTARY MATERIAL

eTable 1. Search strategies used in PubMed and Embase

eTable 2. Quality assessment of the 11 included studies for the meta-analysis

eTable 3. Studies that were excluded from the meta-analysis
