# Supplementary material for: Much Lower Prevalence and Mortality of Chronic Obstructive Pulmonary Disease in Japan Than in the United States Despite Higher Smoking Rates: A Meta-Analysis/Systematic Review
Source: J Epidemiol. 2025 Feb 5;35(2):90–9. doi: 10.2188/jea.JE20240085 (PMC11706673; doi:10.2188/jea.JE20240085)
Supplement: Supplementary file 1 [file je-35-090-s001.pdf]

**eTable 1.** Search strategies used in PubMed and Embase

| Search strategies used in PubMed |
| --- |
| (((((((((((((((((((((((((((((((((((((((((((((((((((((((((((((((((((((((((Hokkaido[Title/Abstract]) OR Hokkaido[Other Term])) OR ((Tohoku[Title/Abstract]) OR Tohoku[Other Term])) OR ((Kanto[Title/Abstract])) OR ((Chubu[Title/Abstract])) OR ((Kansai[Title/Abstract])) OR ((Chugoku[Title/Abstract])) OR ((Shikoku[Title/Abstract])) OR ((Kyushu[Title/Abstract]) OR Kyushu[Other Term])) OR ((Aomori[Title/Abstract])) OR ((Iwate[Title/Abstract]) OR Iwate[Other Term])) OR ((Miyagi[Title/Abstract])) OR ((Akita[Title/Abstract]) OR Akita[Other Term])) OR ((Yamagata[Title/Abstract]) OR Yamagata[Other Term])) OR ((Fukushima[Title/Abstract]) OR Fukushima[Other Term])) OR ((Ibaraki[Title/Abstract]) OR Ibaraki[Other Term])) OR ((Tochigi[Title/Abstract])) OR ((Gunma[Title/Abstract])) OR ((Saitama[Title/Abstract])) OR ((Chiba[Title/Abstract]) OR Chiba[Other Term])) OR ((Tokyo[Title/Abstract]) OR Tokyo[Other Term])) OR ((Kanagawa[Title/Abstract]) OR Kanagawa[Other Term])) OR ((Niigata[Title/Abstract]) OR Niigata[Other Term])) OR ((Toyama[Title/Abstract]) OR Toyama[Other Term])) OR ((Ishikawa[Title/Abstract]) OR Ishikawa[Other Term])) OR ((Fukui[Title/Abstract]) OR Fukui[Other Term])) OR ((Yamanashi[Title/Abstract])) OR ((Nagano[Title/Abstract])) OR ((Gifu[Title/Abstract])) OR (((Shizuoka[Title/Abstract]))) OR (((Aichi[Title/Abstract]) OR Aichi[Other Term]))) OR (((Mie[Title/Abstract]) OR Mie[Other Term]))) OR (((Shiga[Title/Abstract]) OR Shiga[Other Term]))) OR (((Kyoto[Title/Abstract]) OR Kyoto[Other Term]))) OR (((Osaka[Title/Abstract]) OR Osaka[Other Term]))) OR ((Hyogo[Title/Abstract]) OR Hyogo[Other Term])) OR ((Nara[Title/Abstract])) OR ((Wakayama[Title/Abstract])) OR ((Tottori[Title/Abstract])) OR ((Shimane[Title/Abstract])) OR ((Okayama[Title/Abstract]) OR Okayama[Other Term])) OR ((Hiroshima[Title/Abstract]) OR Hiroshima[Other Term])) OR ((Yamaguchi[Title/Abstract]) OR Yamaguchi[Other Term])) OR ((Tokushima[Title/Abstract]) OR Tokushima[Other Term])) OR ((Kagawa[Title/Abstract])) OR ((Ehime[Title/Abstract])) OR ((Kochi[Title/Abstract]) OR Kochi[Other Term])) OR ((Fukuoka[Title/Abstract]) OR Fukuoka[Other Term])) OR ((Saga[Title/Abstract]) OR Saga[Other Term])) OR ((Nagasaki[Title/Abstract]) OR Nagasaki[Other Term])) OR ((Kumamoto[Title/Abstract]) OR Kumamoto[Other Term])) OR ((Oita[Title/Abstract]) OR Oita[Other Term])) OR ((Miyazaki[Title/Abstract]) OR Miyazaki[Other Term])) OR ((Kagoshima[Title/Abstract]) OR Kagoshima[Other Term])) OR ((Okinawa[Title/Abstract]) OR Okinawa[Other Term]))) OR Japan[MeSH Terms]) OR Tokyo[MeSH Terms]) OR Japan[Affiliation]) OR ((Japan[Title/Abstract]) OR Japan[Other Term]))) OR Yokohama[Title/Abstract]) OR Yokohama[Other Term]) OR Nagoya[Title/Abstract]) OR Nagoya[Other Term]) OR Sapporo[Title/Abstract]) OR Kobe[Title/Abstract]) OR Kobe[Other Term]) OR Kawasaki[Title/Abstract]) OR Kawasaki[Other Term]) OR Sendai[Title/Abstract]) OR Sendai[Other Term]) OR Kitakyushu[Title/Abstract]) OR Sakai[Title/Abstract]) OR Sakai[Other Term]) OR Hamamatsu[Title/Abstract]) OR Hamamatsu[Other Term]) OR Sagamihara[Title/Abstract])) OR Japanese[Title/Abstract]) OR Japanese[Other Term]))<br>AND<br>(((((((((((((((((((((((((((Pulmonary Disease, Chronic Obstructive[MeSH Terms]) OR Bronchitis, Chronic[MeSH Terms]) OR Pulmonary Emphysema[MeSH Terms]) OR Bronchitis[MeSH Terms]) OR Bronchiolitis[MeSH Terms]) OR Bronchiolitis Obliterans[MeSH Terms]) OR Cryptogenic Organizing Pneumonia[MeSH Terms]) OR Bronchiolitis, Viral[MeSH Terms]) |

OR Lung Diseases, Obstructive[MeSH Terms]) OR COPD[Title/Abstract]) OR COPD[Other Term]) OR ((Obstructive Pulmonary Diseas*[Title/Abstract]) OR Obstructive Pulmonary Diseas*[Other Term])) OR COAD[Title/Abstract]) OR COAD[Other Term]) OR ((Chronic Obstructive Airway Disease*[Title/Abstract]) OR Chronic Obstructive Airway Disease*[Other Term])) OR ((Obstructive Lung Disease*[Title/Abstract]) OR Obstructive Lung Disease*[Other Term])) OR ((Chronic Airflow Obstruction*[Title/Abstract]) OR Chronic Airflow Obstruction*[Other Term])) OR ((Emphysema*[Title/Abstract]) OR Emphysema*[Other Term])) OR Bronchitides[Title/Abstract]) OR ((Bronchioliti*[Title/Abstract]) OR Bronchioliti*[Other Term])) OR ((Cryptogenic Organizing Pneumonia*[Title/Abstract]) OR Cryptogenic Organizing Pneumonia*[Other Term])) OR ((((Bronchiolitis Obliterans Organizing Pneumonia[Title/Abstract]) OR Bronchiolitis Obliterans Organizing Pneumonia[Other Term])) OR ((Bronchiolitis Obliterans Organizing Pneumonias[Title/Abstract]) OR Bronchiolitis Obliterans Organizing Pneumonias[Other Term]))) OR ((Viral Bronchiolitis[Title/Abstract]) OR Viral Bronchiolitis[Other Term])) OR Chronic Bronchitis[Title/Abstract]) OR Chronic Bronchitis[Other Term]) OR BOOP[Title/Abstract]) OR BOOP[Other Term])))
AND
(((((diagnosis[MeSH Subheading] OR forced expiratory volume[MeSH Terms] OR respiratory function tests[MeSH Terms] OR severity of illness index[MeSH Terms] OR spirometry[MeSH Terms] OR vital capacity[MeSH Terms] OR Diagnose*[Title/Abstract] OR Diagnose*[Other Term] OR Forced Expiratory Volume[Title/Abstract] OR Peak Expiratory Flow[Title/Abstract] OR Forced Expiratory Flow[Title/Abstract] OR Expiratory Flow Rate[Title/Abstract] OR Expiratory Flow Rates[Title/Abstract] OR Forced Vital Capacity[Title/Abstract] OR FVC[Title/Abstract] OR FEV[Title/Abstract] OR FEV1[Title/Abstract] OR GOLD[Title/Abstract] OR Spirometry[Title/Abstract] OR Forced Expiratory Volume[Other Term] OR Peak Expiratory Flow[Other Term] OR Forced Expiratory Flow[Other Term] OR Expiratory Flow Rate[Other Term] OR Expiratory Flow Rates[Other Term] OR Forced Vital Capacity[Other Term] OR FVC[Other Term] OR FEV[Other Term] OR FEV1[Other Term] OR GOLD[Other Term] OR Spirometry[Other Term] OR Tomography[Title/Abstract] OR Tomography[Other Term]))))
AND
((epidemiology[MeSH Subheading] OR Prevalence[MeSH Terms] OR mass screening[MeSH Terms] OR Prevalence[Title/Abstract] OR Prevalent*[Title/Abstract] OR Epidemiolog*[Title/Abstract] OR Epidemiology[Title/Abstract] OR Prevalence[Other Term] OR Prevalent*[Other Term] OR Epidemiolog*[Other Term] OR Epidemiology[Other Term] OR screen*[Title/Abstract] OR screen*[Other Term])))

Search strategies used in Embase

Search:
(('japan'/exp OR 'japan' OR 'japanese (people)'/exp OR 'japanese (people)' )
OR
('hokkaido':ab,ti OR 'tohoku':ab,ti OR 'kanto':ab,ti OR 'chubu':ab,ti OR 'kansai':ab,ti OR 'chugoku':ab,ti OR 'shikoku':ab,ti OR 'kyushu':ab,ti OR 'aomori':ab,ti OR 'iwate':ab,ti OR 'miyagi':ab,ti OR 'akita':ab,ti OR 'yamagata':ab,ti OR 'fukushima':ab,ti OR 'ibaraki':ab,ti OR 'tochigi':ab,ti OR 'gunma':ab,ti OR 'saitama':ab,ti OR 'chiba':ab,ti OR 'tokyo':ab,ti OR 'kanagawa':ab,ti OR 'niigata':ab,ti OR 'toyama':ab,ti OR 'ishikawa':ab,ti OR 'fukui':ab,ti OR

'yamanashi':ab,ti OR 'nagano':ab,ti OR 'gifu':ab,ti OR 'shizuoka':ab,ti OR 'aichi':ab,ti OR 'mie':ab,ti OR 'shiga':ab,ti OR 'kyoto':ab,ti OR 'osaka':ab,ti OR 'hyogo':ab,ti OR 'nara':ab,ti OR 'wakayama':ab,ti OR 'tottori':ab,ti OR 'shimane':ab,ti OR 'okayama':ab,ti OR 'hiroshima':ab,ti OR 'yamaguchi':ab,ti OR 'tokushima':ab,ti OR 'kagawa':ab,ti OR 'ehime':ab,ti OR 'kochi':ab,ti OR 'fukuoka':ab,ti OR 'saga':ab,ti OR 'nagasaki':ab,ti OR 'kumamoto':ab,ti OR 'oita':ab,ti OR 'miyazaki':ab,ti OR 'kagoshima':ab,ti OR 'okinawa':ab,ti OR 'japan':ab,ti OR 'yokohama':ab,ti OR 'nagoya':ab,ti OR 'sapporo':ab,ti OR 'kobe':ab,ti OR 'kawasaki':ab,ti OR 'sendai':ab,ti OR 'kitakyushu':ab,ti OR 'sakai':ab,ti OR 'hamamatsu':ab,ti OR 'sagamihara':ab,ti OR 'japanese*':ab,ti))
AND
(('chronic obstructive lung disease'/exp OR 'chronic obstructive lung disease' OR 'chronic bronchitis'/exp OR 'chronic bronchitis' OR 'emphysema'/exp OR 'emphysema' OR 'bronchitis'/exp OR 'bronchitis' OR 'bronchiolitis'/exp OR 'bronchiolitis')
OR
('copd':ab,ti OR 'obstructive pulmonary diseas*':ab,ti OR 'coad':ab,ti OR 'chronic obstructive airway disease*':ab,ti OR 'obstructive lung disease*':ab,ti OR 'chronic airflow obstruction*':ab,ti OR 'emphysema*':ab,ti OR 'bronchitides':ab,ti OR 'bronchioliti*':ab,ti OR 'cryptogenic organizing pneumonia*':ab,ti OR 'bronchiolitis obliterans organizing pneumonia':ab,ti OR 'bronchiolitis obliterans organizing pneumonias':ab,ti OR 'viral bronchiolitis':ab,ti OR 'chronic bronchitis':ab,ti OR 'boop':ab,ti))
AND
(('epidemiology'/exp OR 'biosurveillance'/exp OR 'biosurveillance' OR 'community assessment'/exp OR 'community assessment' OR 'community sample'/exp OR 'community sample' OR 'cross-sectional study'/exp OR 'cross-sectional study' OR 'disease surveillance'/exp OR 'disease surveillance' OR 'geographic distribution'/exp OR 'geographic distribution' OR 'geographic pathology'/exp OR 'geographic pathology' OR 'health survey'/exp OR 'health survey' OR 'prevalence'/exp OR 'prevalence' OR 'voluntary reporting'/exp OR 'voluntary reporting' OR 'screening test'/exp OR 'screening test' OR 'mass screening'/exp OR 'mass screening' OR 'anonymous testing'/exp OR 'anonymous testing' OR 'developmental screening'/exp OR 'developmental screening' OR 'epidemiology'/exp/dm_dt OR 'epidemiology')
OR
('prevalence':ab,ti OR 'prevalent*':ab,ti OR 'epidemiolog*':ab,ti OR 'epidemiology':ab,ti OR 'screen*':ab,ti))
AND
(('diagnosis'/exp OR 'diagnosis' OR 'severity of illness index'/exp OR 'severity of illness index' OR 'lung volume'/exp OR 'lung volume' )
OR
('diagnose*':ab,ti OR 'forced expiratory volume':ab,ti OR 'peak expiratory flow':ab,ti OR 'forced expiratory flow':ab,ti OR 'expiratory flow rate':ab,ti OR 'expiratory flow rates':ab,ti OR 'forced vital capacity':ab,ti OR 'fvc':ab,ti OR 'fev':ab,ti OR 'fev1':ab,ti OR 'fev 1':ab,ti OR 'gold':ab,ti OR 'spirometry':ab,ti OR 'tomography':ab,ti ))

**eTable 2.** Quality assessment of the 11 included studies for the meta-analysis

| | Fuku-chi 2004[18] | Take-mura 2005[19] | Omori 2007[20] | Mina-kata 2008[21] | Osaka 2010[22] | Horie 2013[23] | Azuma 2014[24] | Fuku-tani 2015[25] | Omori 2016[26] | Utsugi 2016[27] | Omori 2017[28] |
|---|---|---|---|---|---|---|---|---|---|---|---|
| Was the sample frame appropriate to address the target population? | Yes | Yes | Yes | Yes | Yes | Yes | Yes | Yes | Yes | Yes | Yes |
| Were study participants sampled in an appropriate way? | Yes | Yes | Yes | Yes | Yes | Yes | Yes | No | Yes | Yes | Yes |
| Was the sample size adequate? | Yes | Yes | Yes | Yes | Yes | Yes | Yes | No | Yes | Yes | Yes |
| Were the study subjects and the setting described in detail? | Yes | Yes | Yes | Yes | Yes | Yes | Yes | Yes | Yes | Yes | Yes |
| Was the data analysis conducted with sufficient coverage of the identified sample? | Yes | Yes | Yes | Yes | Yes | Yes | N/A | N/A | Yes | Yes | Yes |
| Were valid methods used for the identification of the condition? | Yes | Yes | Yes | Yes | Yes | Yes | Yes | Yes | Yes | Yes | Yes |
| Was the condition measured in a standard, reliable way for all participants? | Yes | Yes | Yes | Yes | Yes | Yes | Yes | Yes | Yes | Yes | Yes |
| Was there appropriate statistical analysis? | Yes | Yes | Yes | Yes | Yes | Yes | Yes | Yes | Yes | Yes | Yes |
| Was the response rate adequate, and if not, was the low response rate managed appropriately? | Unclear | N/A | N/A | N/A | Unclear | N/A | Yes | Unclear | Unclear | N/A | Unclear |

**eTable 3.** Studies that were excluded from the meta-analysis

| Author, Year, ref | # of participants (Male %) | Age Mean (range) | Population drawn | Use of broncho-dilator | FEV1/FCV <70% | Patients with COPD excluded |
|---|---|---|---|---|---|---|
| Kojima 2005[29] | 11,460 (66) | 20-74 | Health checkup | No | Standard procedures | Asthma and tuberculosis |
| Fukahori 2009[30] | 1,424 (46) | 66.0 (40+) | Primary Healthcare | No | ATS | Asthma, pulmonary fibrosis, and lung cancer |
| Kimura 2011[31] | Study I 3,592 (32) Study II 363 (47) | 62.3 | Annual mass-screening Randomly selected volunteers | No | ATS | |
| Muro 2016[32] | 9,040 (33) | 54 | Population-based study | No | Standard procedures | |
| Fukuyama 2016[33] | 2,232 (43) | 63.8 | Population-based study | Yes | JRS guideline | |

ATS, American Thoracic Society; COPD, chronic obstructive pulmonary disease; $FEV_1/FVC$, forced expiratory volume in the first second of the forced vital capacity; JRS, Japanese Respiratory Society.
